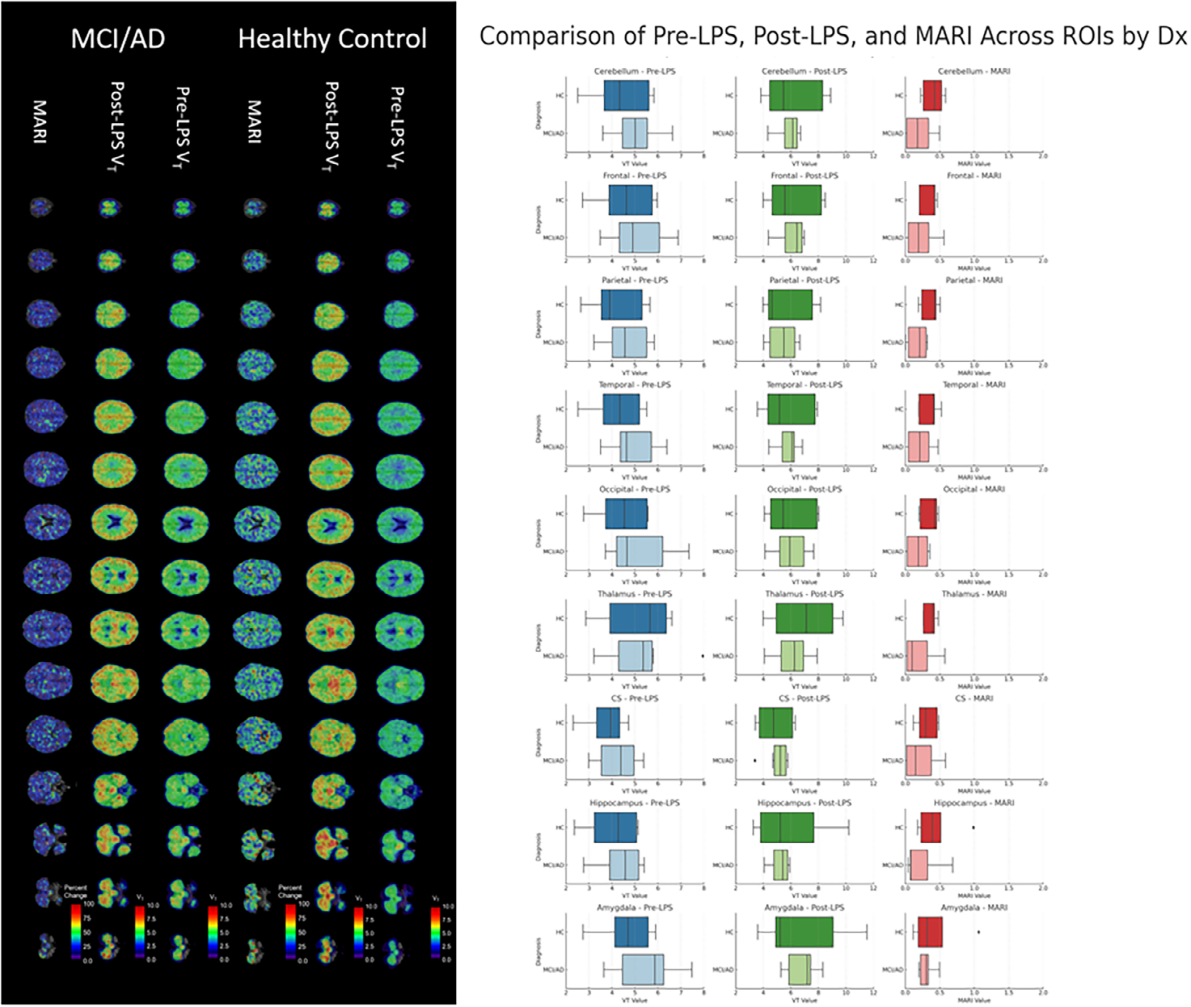# Blunted Microglial Reactivity to Lipopolysaccharide in Alzheimer's Disease: A Novel Insight into Neuroimmune Dysfunction

**DOI:** 10.1002/alz70855_106912

**Published:** 2025-12-24

**Authors:** Arash Salardini, Arsalan Hashemi‐Aghdam, Ryan S O'Dell, Ansel T Hillmer, Daniel F Camacho, Kate M Van Dyck, Kimberly Nelsen, Audrey Ruan, Jessica Lam, Stephen M Strittmatter, Kelly Cosgrove, Christopher H van Dyck, Adam P Mecca

**Affiliations:** ^1^ University of Texas Health Science Center, San Antonio, TX, USA; ^2^ Department of Neurology, University of Texas Health Sciences Center, San Antonio, TX, USA; ^3^ Glenn Biggs Institute for Alzheimer's and Neurodegenerative Diseases, University of Texas Health Science Center, San Antonio, TX, USA; ^4^ Tuft School of Medicine, Boston, MA, USA; ^5^ Yale School of Medicine, New Haven, CT, USA; ^6^ Alzheimer's Disease Research Unit, Yale University School of Medicine, Department of Psychiatry, New Haven, CT, USA; ^7^ Yale Department of Psychiatry, New Haven, CT, USA; ^8^ St. Lawrence University, Canton, NY, USA; ^9^ Alzheimer's Disease Research Unit, Yale School of Medicine, New Haven, CT, USA

## Abstract

**Background:**

Alzheimer's disease (AD) is characterized by chronic neuroinflammation and impaired microglial reactivity, which may underlie the limited efficacy of anti‐inflammatory therapies. Experimental endotoxemia, involving peripheral lipopolysaccharide (LPS) administration, provides a controlled method to assess microglial function in vivo. We investigated whether AD is associated with blunted microglial activation in response to LPS using positron emission tomography (PET) with the translocator protein (TSPO) tracer [11C]PBR28.

**Methods:**

In this study, 12 participants (6 healthy controls [HC], 6 with mild cognitive impairment [MCI] or AD) underwent [11C]PBR28 PET scans before and 3 hours after LPS administration. Microglial activation was quantified using the Microglial Activation Reserve Index (MARI), calculated as the percentage change in regional volume of distribution (*V*T) post‐LPS. Cognitive, demographic, and biomarker data were collected, and safety was monitored through cognitive assessments and adverse event reporting.

**Results:**

The MCI/AD group exhibited a blunted microglial response to LPS compared to HC, as evidenced by a trend toward significance in MARI (Welch's t‐test, *p* = 0.064, Cohen's d = 1.26). Robust regression analysis adjusting for covariates (APOE genotype, sex, age, and scan interval) confirmed a significant association between diagnostic status and MARI (β = ‐0.1196, *p* = 0.041). However, bootstrapped confidence intervals suggested instability in the model due to the small sample size. No significant cognitive or clinical adverse effects were observed post‐LPS administration.

**Conclusions:**

These findings suggest a paradoxical impairment in microglial reactivity to immune challenges in AD, despite the literature showing an elevated baseline neuroinflammation. This novel observation challenges current models of neuroimmune dysfunction in AD and supports the development of therapies aimed at restoring microglial function rather than broadly suppressing inflammation. Future studies with larger cohorts are needed to confirm these results and explore their therapeutic implications.